# Adult single-system cutaneous Langerhans cell histiocytosis presenting with hypopigmented lesions in skin of color: A case report and literature review

**DOI:** 10.1016/j.jdcr.2026.08.059

**Published:** 2026-09-23

**Authors:** Amira Muftah, Zachary Dwyer, Adnan Mansoor, Jori Hardin

**Affiliations:** a Division of Dermatology, Department of Medicine, University of Calgary, Calgary, Alberta, Canada; b Department of Pathology & Laboratory Medicine, University of Calgary, Calgary, Alberta, Canada

**Keywords:** hypopigmentation, Langerhans cell histiocytosis, skin of color

## Introduction

Langerhans cell histiocytosis (LCH) is a rare, clonal proliferative disorder of CD207+ myeloid dendritic cells, known as Langerhans cells, which can affect various organs including the bones, bone marrow, skin, lungs, liver, spleen, and central nervous system.^1^^,^^2^ Clinically, LCH varies from single-system (*S-S*) to multi-system (*M-S*) disease.^1^^,^^2^ *S-S* disease refers to involvement limited to a single organ (eg, skin), whereas multi-system disease involves 2 or more organ systems (eg, lung, bone, and central nervous system). Distinguishing between these forms is essential, as systemic involvement carries significant therapeutic and prognostic implications.

LCH pathogenesis involves activating somatic mutations in the protein kinase signaling pathway.^1^ The *BRAF*V600E mutation, identified in up to 50% to 60% of patients, leads to continuous mutations in the protein kinase pathway activation and uncontrolled Langerhans cell proliferation.^1^ Diagnosis hinges on histopathologic examination and immunophenotyping demonstrating CD1a, S100, and Langerin (CD207) expression.^1^

LCH more commonly affects children, with an estimated incidence of 2 to 10 cases per million.^1^ The prevalence of skin-limited *S-S* LCH is exceedingly rare. In a retrospective study of 274 patients with LCH across 13 countries, skin-limited disease accounted for 7.01% of patients, whereas cutaneous involvement amongst patients with *M-S* LCH was 50.5%.^3^ Cutaneous involvement typically presents as erythematous, pink, skin-colored, or purpuric papules or pustules, predominately involving the trunk, head, or face.^2^ In individuals with skin of color, LCH may present as hypopigmented, scaly papules, macules, patches, or plaques which may mimic more common conditions such as pityriasis alba, pityriasis versicolor, postinflammatory hypopigmentation, vitiligo, or seborrheic dermatitis and may necessitate biopsy when lesions are persistent, atypical, or refractory to therapy.^2^ We report a case of biopsy-proven, *S-S* cutaneous LCH in an adult patient with light brown skin (Fitzpatrick IV phototype), manifesting as scaly, hypopigmented macules and patches with associated erythematous papules.

## Case report

A 48-year-old Filipino woman with a history of migraines and remote *Mycobacterium avium* complex lung infection presented with a 1-year history of recurrent, pruritic, scaly hypopigmented macules and patches with associated erythematous papules involving the face and upper back. Over this 1-year course, the patient underwent initial biopsies 9 months prior to presentation, and at presentation (Fig 1) had repeat biopsies (Fig 2) and systemic evaluation, with subsequent follow-up in our clinic 4 months later.

**Fig 1:**
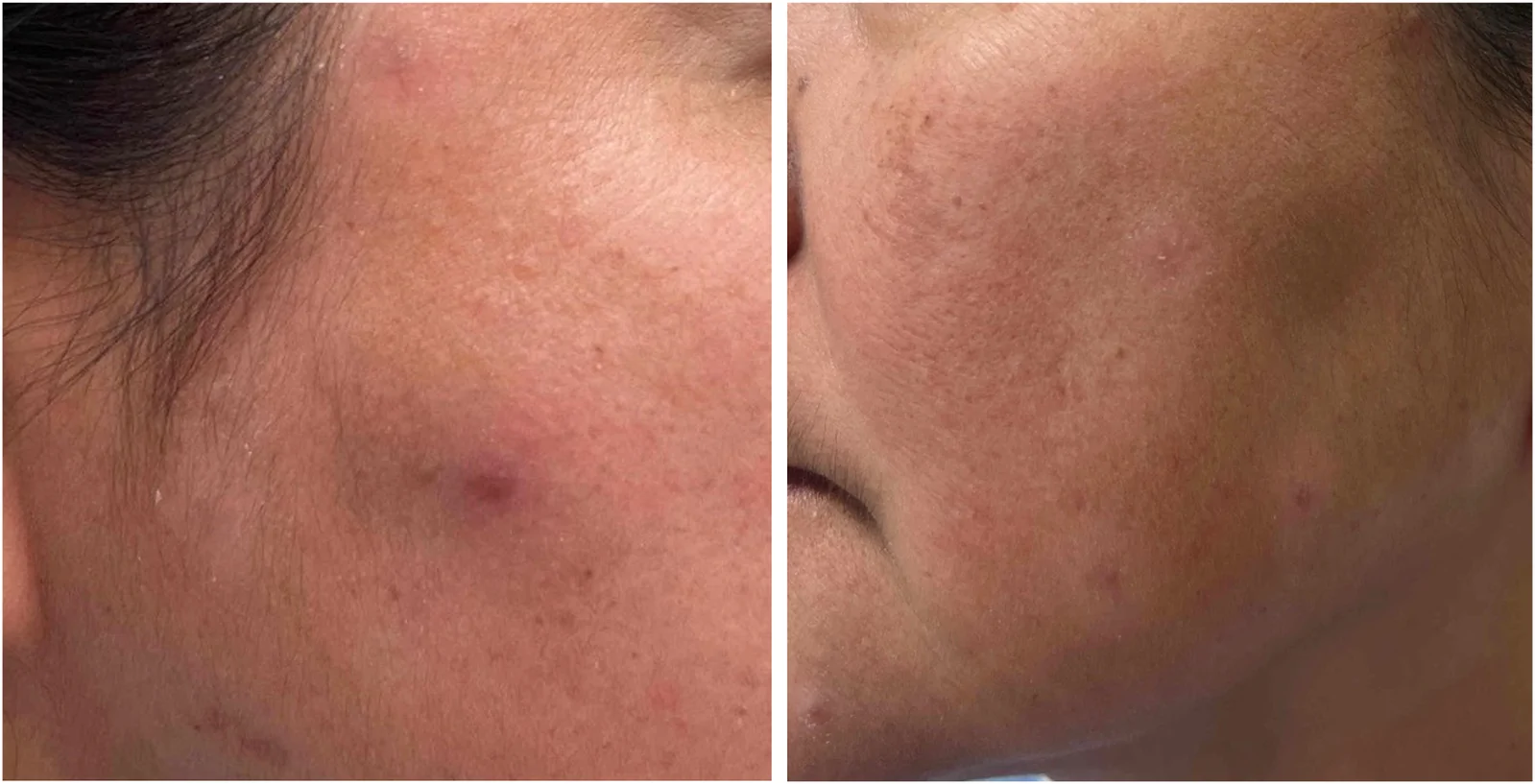
Cutaneous Langerhans cell histiocytosis. Multiple scaly, hypopigmented macules and patches involving the bilateral cheeks and chin, with some lesions showing a distinct central erythematous macule or papule.

**Fig 2:**
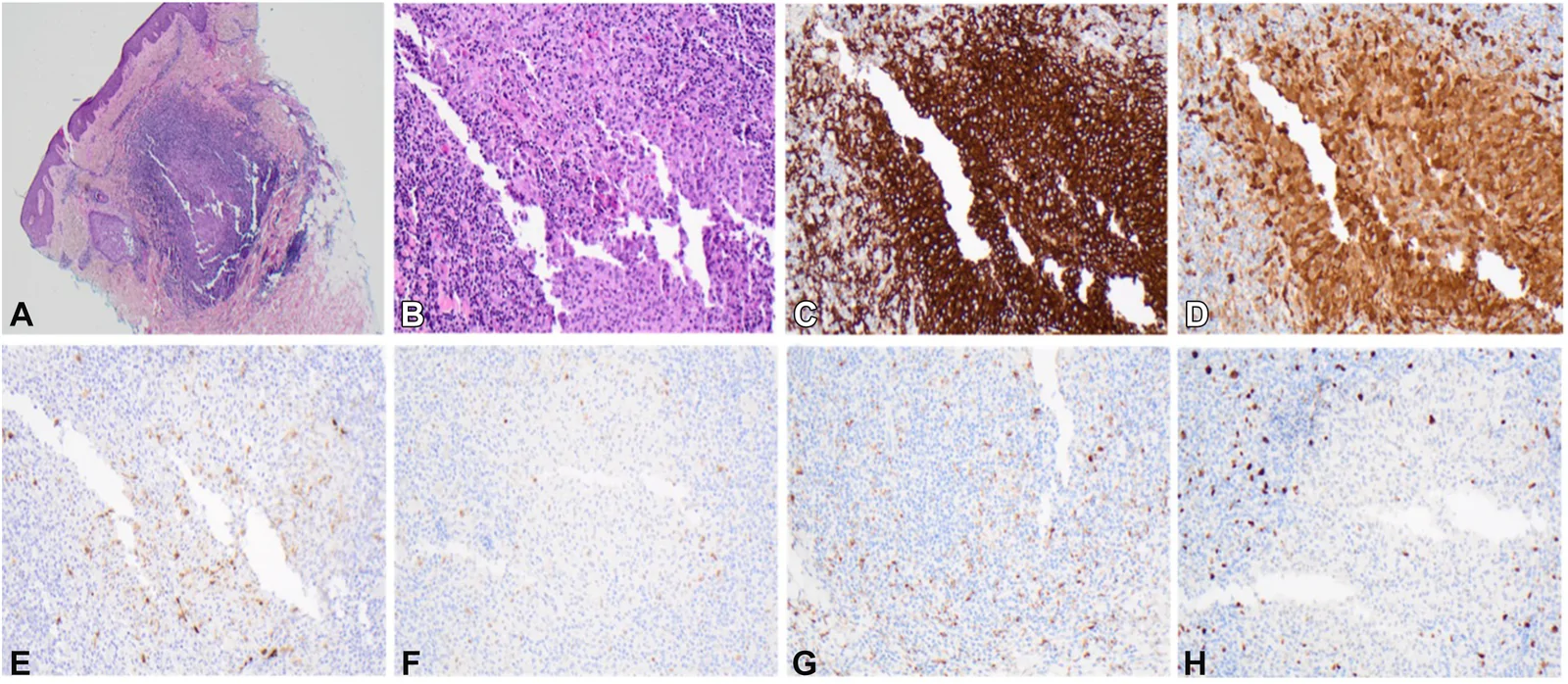
Cutaneous Langerhans cell histiocytosis. Images demonstrate a deep dermal nodule composed of Langerhans cells (**A,** H&E 40×; **B,** H&E 200×) which show strong positivity for CD1a **(C)**, S100 **(D)**, focal CD207 positivity **(E)**, weak Cyclin D **(F)**, granular CD68 **(G)**, and low Ki-67 positivity **(H)**.

At presentation, she described lesion evolution from time of onset. Lesions evolved such that erythematous papules arose from preceding hypopigmented macules and patches, gradually flattened over approximately 2 months, and resolved with residual hyperpigmented macules, while some hypopigmented lesions persisted. Lesions ranged from 10 to 20 mm, were nonfolliculocentric, and involved the face without scalp, genital, mucosal, or nail involvement. Sites of previous biopsies demonstrated scar. No lymphadenopathy was noted.

Histopathologic examination across both biopsies demonstrated a superficial and deep dermal infiltrate composed of lymphocytes, histiocytes, and eosinophils, with nodular aggregates of Langerhans cells (Fig 2). Immunohistochemistry was strongly positive for CD1a and S100, with focal langerin expression and weak Cyclin D1 positivity. The Ki-67 proliferative index was approximately 2% in the initial biopsy and 10% in the subsequent biopsy. *BRAF*V600E mutation testing, performed by polymerase chain reaction (Agena iPLEX HS Melanoma panel, MassARRAY system), was negative. Periodic acid-Schiff stain was negative for fungi. Fite and Ziehl-Neelsen staining was negative for Mycobacterium. The findings were reviewed by a multidisciplinary hematopathology team, and consensus supported a diagnosis of cutaneous LCH.

To rule out *M-S* involvement, the patient underwent a comprehensive workup. complete blood counts, C-reactive protein, liver and renal function tests, electrolytes, thyroid stimulating hormone, lactate dehydrogenase, serum protein electrophoresis, free light chains, and immunoglobulins were all normal. A whole-body fluorodeoxyglucose positron emission tomography scan showed no abnormal uptake.

The review of systems was negative for polyuria, polydipsia, bone pain, headaches, ear symptoms, and visual changes. She reported a 20-pound weight loss over the past year which was evaluated by her primary care provider, with no underlying systemic cause identified and no clear temporal association with her dermatologic course. She denied respiratory symptoms, including cough or dyspnea, and recent chest imaging showed no evidence of active pulmonary disease. Given her remote history of *M avium* complex infection and the possibility of pulmonary LCH, these findings provided additional reassurance in the context of a negative fluorodeoxyglucose positron emission tomography scan.

At 4-month follow-up, a new asymptomatic papule developed on the left lateral neck. Given the indolent course, minimal symptom burden, and absence of systemic involvement, a shared decision was made to pursue observation rather than initiate skin-directed therapy. Treatment options, including topical corticosteroids, were discussed; however, observation was favored given the limited disease extent and lack of symptoms. The patient was counseled that treatment would be reconsidered in the event of increased symptom burden (eg, pruritus, pain, ulceration, or bleeding), disease progression (including development of new lesions), or evidence of systemic disease. Ongoing follow-up was subsequently arranged at regular 12-month intervals for skin and lymph node examination, with repeat imaging to be guided by the development of new symptoms or clinical concern.

## Discussion

LCH is a rare clonal proliferative disorder of CD207+ myeloid dendritic cells, known as Langerhans cells, involving multiple organs.^1^^,^^2^ Clinically, LCH ranges from single-to multi-system disease. Diagnosis relies on histopathology demonstrating CD1a, S100, and Langerin (CD207) expression.^1^ Cutaneous manifestations commonly include erythematous, pink, skin-colored, or purpuric papules or pustules affecting the trunk, head, and face.^2^ Hypopigmented presentations are rare. A comprehensive Ovid MEDLINE search from inception to November 2025, using the terms “Langerhans cell histiocytosis” and “hypopigmentation” and supplemented by manual handsearching of reference lists, identified 24 published cases of hypopigmented cutaneous LCH. Inclusion criteria comprised English-language case reports, case series, and abstracts describing hypopigmented cutaneous LCH across all ages. Key findings are summarized below (Table I).

• Age at presentation: Most reported cases occurred in neonates and infants, often demonstrating spontaneous regression. Five cases were specifically described as congenital self-healing reticulohistiocytosis, representing the self-limited end of the LCH spectrum.^8^^,^^14^^,^^15^^,^^18^^,^^21^ Adult-onset skin-limited LCH presenting with hypopigmented lesions is exceedingly rare, with only 1 previously reported adult case by Bishnoi et al.^19^
• Lesion distribution: Most commonly involved sites include the trunk (22/24), extremities (17/24), scalp (8/24), and face (5/24).
• Histopathology: Consistent with our findings, reported cases demonstrated Langerhans cell proliferation with immunophenotypic positivity for CD1a, S100, and/or Langerin (CD207).
• Presentation of hypopigmentation: Hypopigmentation variably represented the primary manifestation, coexisted with other cutaneous LCH variants as in our case, or reflected resolving disease.
• Race and ethnicity: Many reported cases involved diverse populations, including Asian, African American, and Hispanic individuals.
• Systemic involvement: Nine of 24 cases demonstrated *M-S* involvement, emphasizing the importance of systemic evaluation and ongoing monitoring.^5^^,^^7^^,^^9^^,^^10^^,^^12^^,^^13^^,^^17^^,^^19^^,^^22^

**Table I:** Summary of reported cases of LCH presenting with hypopigmented lesions

Author (year, number of cases)	Location	Age of onset	Sex	Race	Cutaneous findings	Location	Hypopigmentation type	IHC positivity	BRAF	Systemic involvement	Clinical course
Longaker et al^4^ (1994, *n* = 1)	US	3 wk	M	Asian	Vesicopustules, crusted papules, atrophic hypopigmented macules	Trunk, extremities	Primary	S100	NA	None	SR by 11 mo of age
Seth et al^5^ (2006, *n* = 1)	India	10 mo	M	NA	Hypopigmented macules, erythematous, scaly, crusted plaques	Face, trunk, soles	Primary	S100	NA	LN	NA
Raymar et al^6^ (2008, *n* = 1)	Iran	Birth	F	NA	Erythematous papulonodules followed by hypopigmented macules	Trunk, extremities	Secondary	CD1a, S100	NA	None	SR by 9 mo of age
Satter and High^7^ (2008, *n* = 1)	US	3 y	M	NA	Erythematous macules and papules with overlying yellow crust, petechiae, purpura, hypopigmented macules in areas of partial resolution	Scalp, neck, back	Secondary	CD1a, S100	NA	Skull and iliac OL, DI	CR of cutaneous lesions with systemic therapy Ongoing therapy for DI at time of follow-up
Chang et al^8^ (2008, *n* = 1) abstract only	Taiwan	Neonate	F	NA	Hypopigmented macules and papules	Trunk	NA	Positive, unspecified	NA	NA	NA
Feroz et al^9^ (2008, *n* = 1)	India	4 mo	M	NA	Hypopigmented macules	Face, neck, chest, back	Primary and secondary	S100	NA	Skull OL, DI, proptosis	CR of cutaneous lesions with systemic and topical therapies
Aggarwal et al^10^ (2010, *n* = 1)	India	Birth	M	NA	Erythematous-brown papulonodules followed by hypopigmented macules	Entire body including soles and scalp	Secondary	CD1a, S100	NA	Pulmonary	SR of pulmonary and cutaneous lesions by 2 and 6 mo of age, respectively
Battistella et al^11^ (2010, *n* = 4)	France	<15 d (*n* = 4)	NA	NA	Hypopigmented macules	Trunk, extremities	Primary	CD1a	NA	None	SR by 4 mo of age
Kaddu et al^12^ (2010, *n* = 1)	Uganda	7 y	M	African	Hypopigmented macules, erythematous, scaly papules	Scalp, face	Primary	CD1a, S100	NA	Exophthalmos	Lost to follow-up
Mehta and Amladi^13^ (2010 *n* = 1)	India	14 mo	M	NA	Hypopigmented papules	Face, trunk, extremities	Primary	S100	NA	Skull OL	CR of cutaneous lesions and improvement in skull OL with systemic therapy
Shetty et al^14^ (2014, *n* = 1)	India	Birth	F	NA	Erythematous-brown papules followed by hypopigmented macules	Entire body	Secondary	S100, CD68	NA	None	SR within a few mo
Uaratanawong et al^15^ (2014, *n* = 1)	Thailand	2 wk	F	Thai	Hypopigmented flat-topped papules	Trunk, extremities	Primary	CD1a, S100	NA	None	SR by 6 mo of age
Tan et al^16^ (2015, *n* = 1)	China	2 d	M	NA	Crusted erythematous papules, hypopigmented macules	Trunk, extremities, face, scalp	Secondary	CD1a, S100	NA	None	SR by 6 mo of age
Masdemont et al^17^ (2017, *n* = 1)	Spain	2 mo	M	Hispanic	Hypopigmented papules	Trunk, lower limbs	Primary	CD1a, S100 CD207	NA	Skull, humerus, femur OL	CR with systemic therapy
Parimi et al^18^ (2017, *n* = 1)	China	Birth	F	NA	Erythematous-brown papules, hypopigmented macules	Scalp, trunk, groin, extremities	Secondary	CD1a, S100	NA	None	SR by 7 mo of age
Bishnoi et al^19^ (2018, *n* = 1)	India	30 yr	M	NA	Hypopigmented papules, crusted papulopustules, dystrophic nails	Scalp, back	Primary	CD1a	NA	Femoral condyle OL, pulmonary nodules, hypopituitarism DI	CR of skin and bone lesions within 3 mo of topical and systemic therapy, mild fibrotic lung changes and ongoing treatment for DI at time of follow up
Mori et al^20^ (2018, *n* = 2)	USA	5 wk	M	African American	Hypopigmented macules	Scalp, trunk, extremities, groin, buttocks	Primary	CD1a	NA	None	SR by 4 mo of age
		Birth	F	African American	Hypopigmented macules and papules, erythematous plaques and papules	Scalp, back, neck, axillae, groin	Primary	CD1a	Negative	None	SR by 16 mo of age
Wu et al^21^ (2019, *n* = 1)	China	5 mo	F	NA	Hypopigmented papules	Neck, trunk	Primary	CD1a, S100, Langerin (CD207), CD68, Ki-67 (6%)	BRAF V600 E+	None	SR by 10 mo of age
Bencharattanaphakhi et al^22^ (2021, *n* = 1)	Thailand	1 wk	F	NA	Hypopigmented patches	Labia majora	Primary	CD1a, S100, Langerin (CD207), CD68	NA	OL of long bones	CR of cutaneous and boney lesions with systemic therapy
Xiao and Shon^23^ (2024, *n* = 1)	USA	Birth	M	Hispanic	Hypopigmented macules, pink papules	Trunk, inguinal folds	Primary	CD1a, S100	NA	None	SR by 10 mo of age
Current case (2024, *n* = 1)	Canada	48 y	F	Filipino	Scaly hypopigmented macules, erythematous papules	Face, neck, back	Primary	CD1a, S100, Langerin, Cyclin D1, CD68 Ki-67 (2%, 10%)	Negative	None	New papule at 4 mo follow-up

∗Primary hypopigmentation refers to lesions presenting initially as hypopigmented; secondary hypopigmentation refers to hypopigmentation occurring in previously affected areas or resolving lesions.

*CR*, Complete regression; *DI*, diabetes insipidus; *IHC*, immunohistochemistry; *LN*, lymphadenopathy; *NA*, not assessed; *OL*, osteolytic lesions; *SR*, spontaneous regression.

The mechanism underlying hypopigmentation in LCH remains incompletely understood. Reported cases describe hypopigmentation as both a primary finding and a secondary feature of resolving inflammation. A plausible explanation is that Langerhans cell infiltration disrupts melanocyte function, melanogenesis, or melanosome transfer. In our case, lesions evolved from hypopigmented macules to erythematous papules and subsequently to dyspigmentation, suggesting that hypopigmentation may represent an early disease phase preceding clinically apparent inflammation, followed by reactive pigmentary changes.

This case highlights an uncommon presentation of adult single-system cutaneous LCH and underscores the importance of considering LCH in the differential diagnosis of hypopigmented lesions, particularly in skin of color. Although our patient demonstrated an indolent, skin-limited course, ongoing surveillance remains essential, as disease progression or systemic involvement may occur over time. The development of a new lesion at 4-month follow-up in our patient further emphasizes that even in apparently stable cases, close clinical monitoring is warranted. Greater awareness of these heterogeneous presentations can improve early recognition, appropriate investigation for systemic disease, and timely management.

## Conflicts of interest

None disclosed.
